# User acceptance and continuance intention of the BeSt age mHealth application for physical activity promotion and fall prevention in nursing homes

**DOI:** 10.3389/fdgth.2026.1696474

**Published:** 2026-06-23

**Authors:** Jonathan Diener, Jelena Krafft, Kerem Doğan, Iris Ten Klooster, Janina Krell-Roesch, Lisette van Gemert-Pijnen, Alexander Woll, Kathrin Wunsch

**Affiliations:** 1 Institute of Sports and Sports Science, Karlsruhe Institute of Technology, Karlsruhe, Germany; 2 Department of Psychology, Health and Technology, Centre for EHealth and Wellbeing Research, TechMed Centre, Faculty of Behavioural, Management and Social Sciences, University of Twente, Enschede, Netherlands

**Keywords:** continuance intention, fall prevention, mHealth, nursing home, user acceptance

## Abstract

**Background:**

Falls pose a major threat to nursing home residents, highlighting urgent prevention needs. Mobile health applications offer promising solutions, but their effectiveness depends on acceptance and sustained use. Despite increasing attention to digital care technologies, adherence remains low and integration into care practice limited. This study aimed to examine acceptance of the BeSt Age App, a fall prevention mobile application for nursing homes, and to analyze the influence of perceived usefulness and usability on continuance intention.

**Methods:**

A cluster-randomized controlled trial was conducted in nursing homes in Southern Germany. Over 12 weeks, trained nursing home employees used the BeSt Age App to provide individualized exercise sessions to nursing home residents. User acceptance data and related usage constructs were collected from nursing home employees (adherence, usability, user experience, perceived usefulness, engagement, continuance intention) and residents (continuance intention, motivation). Multiple regression was conducted to examine factors influencing continuance intention.

**Results:**

Eleven nursing homes with 37 employees and 137 residents participated. Residents (mean age 85.0 ± 7.6 years, 81% female) showed moderate cognitive impairment and low digital competence (1.9 ± 1.1; scale: 1–5). Employees (mean age 51.7 ± 11.5 years, 84% female, digital competence 3.54 ± 0.8) rated usability with 87.1 ± 15 points and reported positive continuance intention (4.03 ± 1.22; scale: 1–5). Employee adherence was 85.6%, resident adherence 75.1%. Perceived usefulness (*β* = .39, *p* = .020) and usability (*β* = .46, *p* = .047) significantly predicted continuance intention (R² = .377).

**Discussion:**

Nursing home employees evaluated the BeSt Age App positively, particularly with respect to usability, which, alongside perceived usefulness, predicted continuance intention. The digital divide between employees and residents emphasizes the critical role of employee-mediated technology interventions for successful implementation in long-term care. Future research should examine longer-term adoption patterns and investigate the relationship between sustained app usage and clinical outcomes in nursing home settings.

## Introduction

As populations age and the number of individuals residing in nursing homes increases, the challenges associated with physical health problems and the risk of falls necessitate innovative approaches to enhance health outcomes in this setting. Falls among nursing home residents are a significant concern, as these individuals experience 1.7 falls per resident annually compared to 0.65 falls in community-dwelling older adults (1). A Canadian study reported that 62% of nursing home residents experienced at least one fall over the course of a year (2). These numbers highlight the urgent need for effective fall prevention strategies, as falls can have severe health consequences (3).

One of the most promising approaches to reducing fall risk is promoting regular physical activity, which plays a crucial role in mitigating age-related physical decline in physical and cognitive function, preserving mobility, and supporting the overall independence of nursing home residents (4). To promote healthy aging, the International Association of Gerontology and Geriatrics recommends multicomponent training for nursing home residents, focusing on strength, aerobic endurance, balance, and flexibility, at least twice per week (5). Exercise is known to significantly enhance functional performance, strength, and balance, all of which are critical in reducing fall risk (6). Digital solutions, such as mobile health (mHealth) applications, may offer a promising way to deliver the aforementioned interventions. For example, they can support nursing home employees in providing personalized physical activity and fall prevention programs tailored to residents' capabilities and needs (7). These tools can assist care providers in overcoming barriers to engaging in physical activity, such as lack of motivation or knowledge of appropriate exercises (8).

However, a gap often exists between the theoretical potential of technology and its implementation in practice (9), as shown by low adherence rates observed in several studies (10). For mHealth technologies to deliver meaningful benefits in care settings, they must first be accepted by their intended users and subsequently integrated into daily practice (11). Acceptance refers to the decision to make use of a technology and is a prerequisite for successful implementation (9). Importantly, acceptance is not a static outcome but a dynamic process. Perceptions of acceptability may emerge before use (pre-use acceptability), evolve during usage (concurrent acceptability), and be reassessed after a period of use (retrospective acceptability) (12). This process-oriented view of acceptance helps explain how initial receptivity develops into sustained usage decisions.

The Technology Acceptance Model (TAM) and its extensions (TAM2, TAM3) conceptualize initial user acceptance primarily through perceived usefulness and perceived ease of use, which shape intention to adopt a new system (13–15). Post-adoption models, such as the Post-Acceptance Model of Information Systems Continuance (16), extend this perspective by examining how perceptions evolve during real-life use. Continuance intention can be understood as a construct closely related to retrospective acceptability, reflecting experience-based evaluations of usefulness and satisfaction. In the present study, we examined how perceived usefulness and usability influence continuance intention. To provide a more comprehensive understanding of the user acceptance process, we examined additional constructs. User experience (UX), referring to users' cognitive and affective responses to interacting with technology, has been shown to influence satisfaction and perceived value—key drivers of continuance intention (17). Engagement is conceptualized as a multidimensional construct comprising behavioral, cognitive, and affective components; it reflects the quality of users' interaction with digital health technologies and is central to understanding how and why individuals sustain use (58). Adherence, representing the extent to which actual use matches the intended or recommended usage pattern, provides an objective behavioral indicator (18). While adherence reflects observed behavior, continuance intention reflects a forward-looking commitment to use.

Most mHealth evaluations have focused on direct users, such as patients or community-dwelling older adults, while giving limited attention to healthcare workers as indirect but crucial adopters in nursing homes (19). At the same time, digital physical activity interventions in nursing homes have so far concentrated mainly on exergaming, which often excludes residents with advanced cognitive or physical impairments (20). This underscores the need to study employee-mediated mHealth solutions that can adapt to diverse functional levels and enable broader participation, bridging the gap between technological potential and practical implementation.

The BeSt Age App was developed to address this gap. It is a tablet-based mHealth application that supports nursing home employees in delivering structured, individualized physical activity sessions for fall prevention. Rather than requiring independent use by residents, the app enables staff to tailor and guide exercises according to residents' functional abilities and preferences, thereby supporting implementation in routine nursing home care.

We evaluated the BeSt Age App over a three-month intervention period in nursing homes. The primary research question was whether perceived usefulness and usability predict nursing home employees’ intention to continue using the app after the intervention. Based on TAM and post-adoption acceptance models, we hypothesized that higher perceived usefulness and higher usability would be associated with stronger continuance intention. In addition, we examined user experience, engagement, and adherence among employees, as well as residents’ adherence and intention to continue participating in the program, to provide a broader understanding of acceptance and implementation in the nursing home context.

## Methods

This study was preregistered in the German National Register of Clinical Trials (DRKS00032349). Ethical approval was obtained from the ethics committee of the Karlsruhe Institute of Technology, Karlsruhe, Germany. A cluster-randomized controlled trial was conducted in nursing homes in Southern Germany. Nursing homes were randomly assigned to either the intervention group or the control group. The rationale, development process, and the study protocol for the BeSt Age intervention can be found in Krell-Roesch et al. (21). This study focuses on the implementation of the BeSt Age App in the intervention group.

### Participants

Nursing homes in the city of Karlsruhe and the surrounding districts in the state of Baden-Wuerttemberg, Germany were recruited through telephone outreach and on-site informational meetings. We included nursing home residents who met the following criteria: (1) age above 65 years, (2) at least 50% functional capacity of the extremities (i.e., at least two of four limbs: both arms, both legs, or one arm and one leg), and (3) ability to follow instructions required for participation in the exercise sessions. Residents were excluded if cognitive, neurological, or motor impairments were so severe that they were unable to safely participate in the group-based exercise program. No single standardized cut-off score was used for this exclusion criterion. Instead, eligibility was assessed by trained study staff during baseline assessment and, where necessary, in consultation with nursing home employees familiar with the residents' everyday functioning. Nursing home employees were eligible to participate if they were aged 18 years or older. Eligibility of participants was verified at baseline assessment based on these inclusion and exclusion criteria. Written informed consent was obtained from all participants or their legal guardians prior to the study.

### Intervention

The intervention was carried out using the tablet-based BeSt Age App. This app was developed through a multi-step process based on an intervention-mapping approach (22), involving theoretical considerations, a literature review (20), an online survey with nursing homes (23), and an interview study with relevant stakeholders. In a two-week pilot study conducted in two nursing homes, the app prototype was tested to evaluate feasibility, usability, and user experience. The app design was informed by behavioral and motivational frameworks, including Social Cognitive Theory (24), Self-Determination Theory (25), and the COM-B model (26), to ensure it was evidence-based, comprehensive, and capable of fostering intrinsic motivation through autonomy, competence, and relatedness.

**Figure 1:**
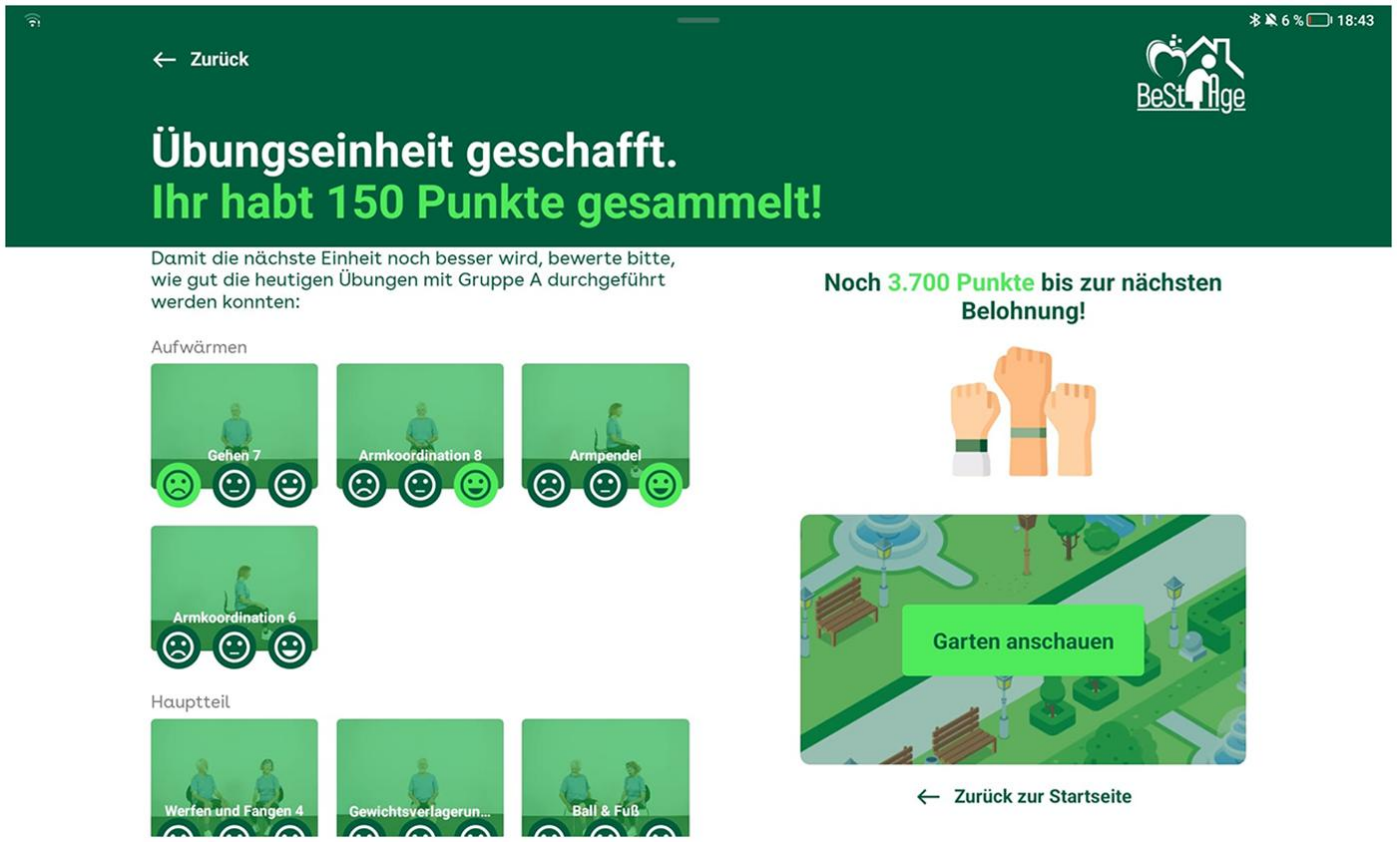
Customization feature. By clicking on the “x” icon, exercises were removed, by selecting the “+” icon, an additional exercise was added. By clicking on the double-arrow icon, the exercise was replaced by a new exercise.

**Figure 2:**
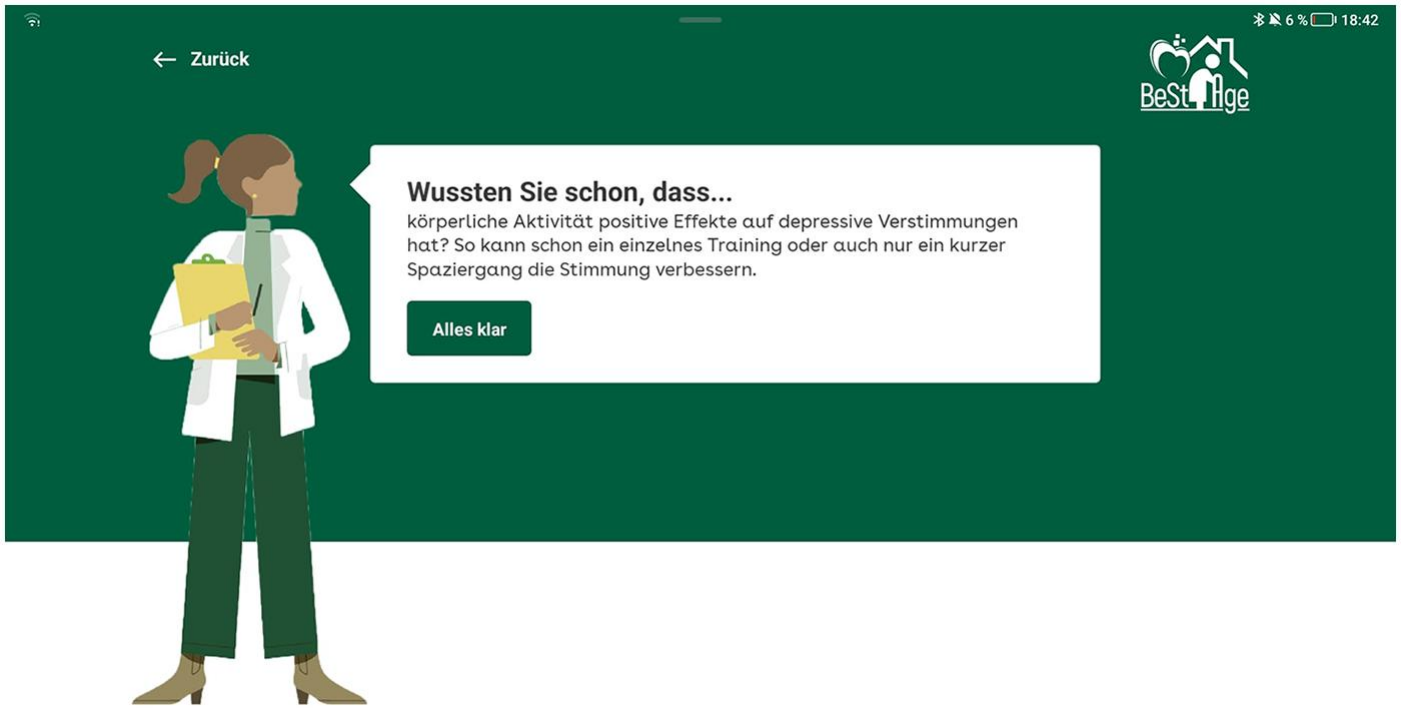
Health literacy feature. Translation: “Did you know that… physical activity can have positive effects on depressive moods? Even a single workout or just a short walk can help improve your mood.”

**Figure 3:**
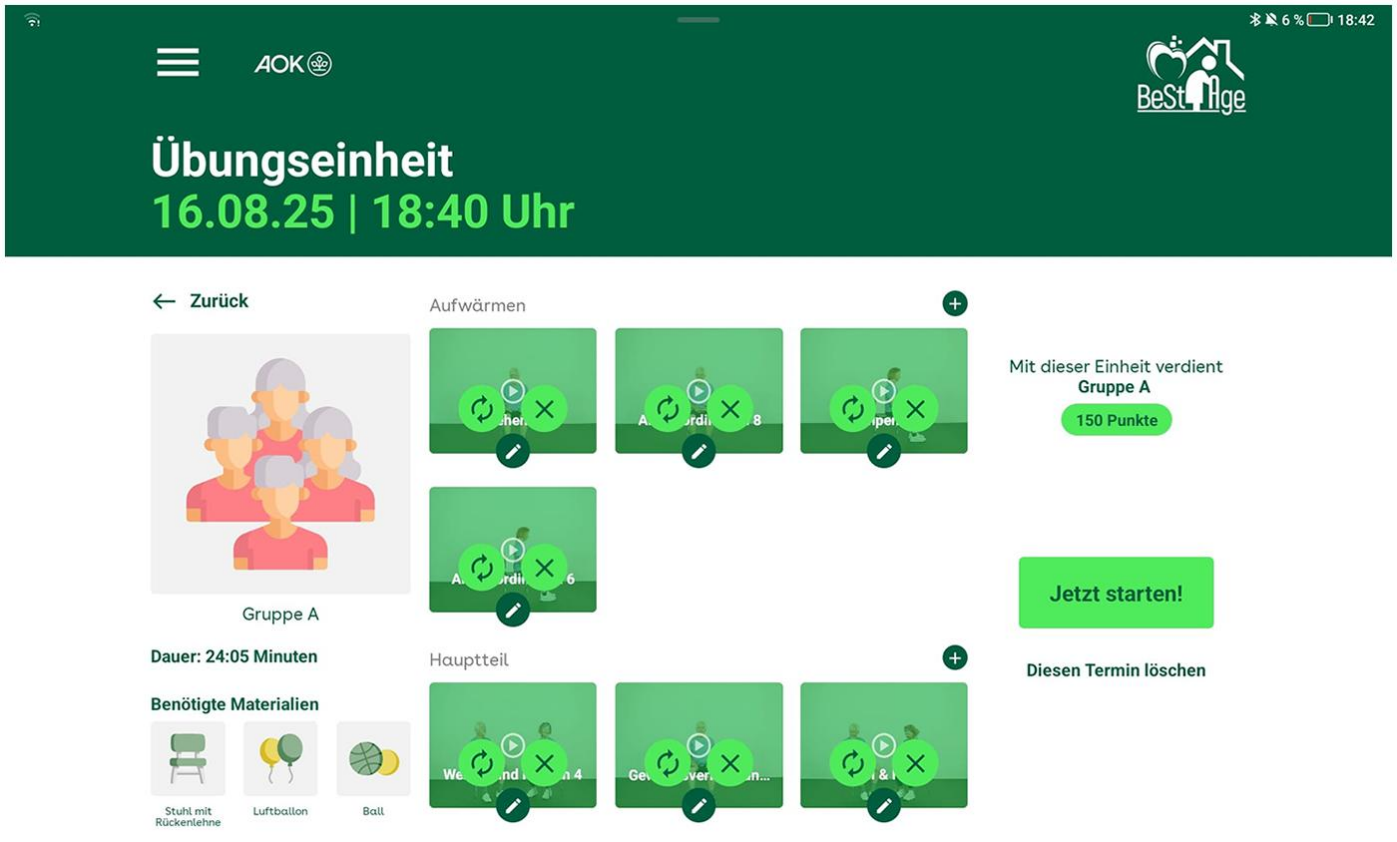
Personalization feature. By clicking on the emojis displayed, employees were able to rate exercises.

The purpose of the app is to assist nursing home employees in providing individualized (i.e., adapted to cognitive and motor abilities) and varied (through a large pool of exercises) exercise sessions. Additionally, the app aims to reduce the workload of the employees by providing fully prepared exercise sessions. To this end, employees created a profile for each resident capturing three categories of information: (1) motor performance level (e.g., ability to stand and walk with or without aids), (2) cognitive performance level (no or mild, moderate, or severe impairment), and (3) personal exercise preferences (e.g., exercising with music or balls). These inputs were entered as structured categorical selections by nursing home employees based on their familiarity with residents' everyday functioning. To maintain feasibility in routine nursing home practice, this pragmatic approach was chosen instead of requiring standardized assessments for each resident, which would have placed considerable additional time demands on employees. Full details of the profiling and matching procedures are provided in the study protocol (21). Once the resident profiles are created, matching algorithms are used to form small, homogeneous groups of up to seven residents with similar motor and cognitive performance levels and exercise preferences. The algorithm matched resident characteristics with the requirements defined for each of approximately 150 available exercises. Employees use the calendar function to schedule exercise sessions twice a week. On the day of the exercise session, employees operate the tablet and deliver the sessions to small groups of residents. The groups are provided with exercises matched with their abilities and interests. If needed, employees can adapt the sessions by removing or adding exercises (customization feature, see Figure 1). Prior to each session, a brief health fact highlighting the risks of physical inactivity and the benefits of regular activity was provided to employees, who were instructed to read it aloud to residents to promote health awareness (health literacy feature, see Figure 2). Following each session, employees rated the exercises using an integrated feedback system, taking into account their own observations as well as residents' verbal and nonverbal feedback. Ratings reflected whether the exercise intensity was appropriate. Exercises that were rated positively several times prompted the algorithm to suggest more challenging progressions in subsequent sessions, whereas poorly rated exercises were excluded from future recommendations for that group (personalization feature, see Figure 3).

**Figure 4:**
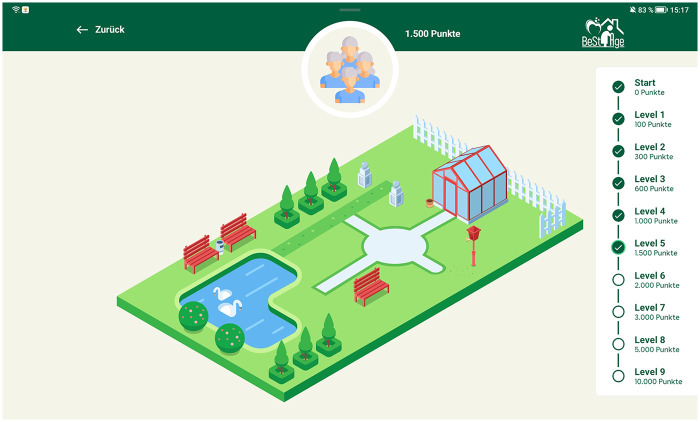
Gamification feature. Points were awarded for completed exercise sessions, which users could redeem for virtual items to personalize the digital nursing home garden.

To enhance motivation, the app incorporated gamification elements, awarding points for completed sessions. These points could be redeemed for virtual items to decorate a digital nursing home garden (gamification feature, see Figure 4). Employees were instructed to show residents the evolving garden on the tablet to engage them and illustrate the impact of their participation.

The intervention was conducted over a continuous 12-week period, with two 30 min sessions per week. Each session consisted of a warm-up, a main part, and a cool-down. The warm-up included exercises for mobilization and coordination, the main part comprised exercises for strength, balance, endurance, and coordination, and the cool-down included exercises for relaxation. Prior to the intervention, all participating employees received standardized on-site training delivered by the same study coordinator to ensure protocol adherence and to account for varying levels of digital competence. The training lasted approximately 1.5–2 h and covered both the technical use of the app as well as strategies to motivate residents before and during the sessions. Employees were instructed to navigate through the app independently and practiced creating test resident profiles and test exercise sessions. After the training, employees received a manual summarizing all relevant procedures and app functions discussed during the session.

### Outcomes

Survey data was collected from nursing home employees as operators of the app, as well as from nursing home residents as the primary target group. Additionally, log data was analyzed. While survey data captured subjective perceptions of the application and the intervention, log data contextualized and enriched these insights by providing objective measures of actual use.

#### Nursing home employees

Descriptive variables included participants' age, sex, job title, years of experience in delivering physical activity programs, certification status for delivering physical activity, and digital competence using a five-item Likert scale ranging from 1 (“Very low”) to 5 (“Very good”).

Post-intervention, usability was evaluated using the System Usability Scale [SUS (27);], scored from 0 to 100, and user experience was assessed with the AttrakDiff Questionnaire (28), ranging from −3 to 3. Higher values on both scales reflect more positive user perceptions. Perceived usefulness was assessed with the question “Do you feel that using the BeSt Age App has made your work easier?” using a 5-Item Likert scale from 1 (not at all) to 5 (completely). Continuance intention was assessed with the question “Would you like to continue using the BeSt Age App after the end of the intervention?” using a 5-item Likert scale from definitely not (1) to definitely yes (5).

#### Nursing home residents

Descriptive variables included participants' age, sex, body mass index (BMI), cognitive status, functional mobility, and digital competence. Cognitive status was assessed using the German version of the Montreal Cognitive Assessment [MoCA (29, 30);], and functional mobility was measured with the Timed Up and Go Test [TUG (31);]. Digital competence was evaluated using a five-item Likert scale ranging from 1 (“Very low”) to 5 (“Very good”).

Post-intervention, continuance intention was assessed via a single question “Would you like to continue participating in the BeSt Age program?” using a 5-item Likert scale, from 1 (definitely not) to 5 (definitely yes). The influence of the gamification feature on motivation to participate in the program was evaluated using a 5-item Likert scale from 1 (not influenced at all) to 5 (strongly influenced).

#### Log data

All user interactions within the app were systematically logged (32) throughout the three-month intervention period, regardless of which employee accessed it via the shared tablet. The system automatically tracked timestamped user interactions including event types (touch, create, update), event targets [e.g., edit profile/add exercise (touch), resident profile (create or update)], group ID, resident ID, exercise ID, and exercise session ID. Usage sessions were defined as continuous periods of app activity, with a new session initiated when user actions occurred after 15 min of inactivity. Prior to study commencement, informed consent for data logging was obtained from all participating employees and residents, with explicit disclosure of the types of interactions to be recorded and assurance of data anonymization and confidentiality in accordance with institutional ethics guidelines.

Log data represent a common source of objective usage measures in mHealth research (33). Short et al. (34) proposed the FITT framework (Frequency, Intensity, Time, and Type) to structure log data analyses, an approach adapted from physical activity research (35). The FITT parameters were defined as follows: (1) Frequency represented the total number of distinct days employees accessed the application during the three-month intervention period. This metric captured the consistency of usage by counting calendar days on which at least one interaction with the app was recorded, regardless of session duration or activity intensity. (2) Intensity was quantified by the aggregate number of screen touches recorded during app sessions. This provided a quantitative measure of user activity level within the application. (3) Time reflected the duration in days spanning from initial login to final recorded session, reflecting persistence of use. (4) Type encompassed the number of touches within the specific application features (personalization, customization, health literacy and gamification), allowing analysis of which functional areas of the application were used most frequently and comprehensively. While Frequency and Intensity are best understood as indicators of user involvement—reflecting how often and how much the app was used—Time and particularly Type provide a closer approximation of engagement, as they capture persistence and the qualitative nature of interaction.

In line with the recommendations for operationalizing adherence in eHealth interventions by Sieverink et al. (18), employee adherence was defined as the percentage of exercise sessions delivered out of the total 24 predetermined sessions and was calculated based on the log data. The intended use for employees was defined *a priori* as delivering all 24 scheduled exercise sessions over the three-month intervention period, with this threshold established based on the study protocol's theoretical framework for achieving desired health outcomes among residents. Adherence of residents, along with reasons for non-adherence, were documented within the app by employees. Resident adherence was measured by calculating the percentage of attendance across the 24 predetermined sessions, with intended use defined as participation in all available exercise sessions. Reasons for resident non-adherence were documented to distinguish between different types of non-usage and provide context for adherence patterns. Additionally, an adjusted adherence rate for residents was computed, representing the percentage of sessions attended relative to the number of sessions actually delivered, thereby accounting for any sessions that were not conducted due to employee-related factors. To examine adherence trajectories over time, the intervention period was divided into three equal intervals of four weeks each (weeks 1–4, 5–8, and 9–12). Additionally, adherence rates were aggregated at the nursing home level to assess between-facility variability.

### Statistical analysis

All statistical analyses were performed using SPSS version 29 (IBM Corp) with alpha (*α*) set *a priori* at 0.05. Descriptive statistics (means, standard deviations, frequencies) were calculated to summarize sample characteristics and key variables. The sample size was determined in the context of the broader cluster-randomized controlled trial; full details of the *a priori* power calculation are reported in the study protocol (21). The present paper reports exclusively on the intervention group, as technology acceptance and app usage outcomes were only assessed in participants who used the BeSt Age App. No separate power analysis was conducted for these outcomes, which should be considered when interpreting the present findings. Missing data were handled using listwise deletion given the low number of incomplete cases. A multiple regression analysis was conducted to examine the influence of usability and perceived usefulness on continuance intention. Homoscedasticity was assessed using the Breusch-Pagan test. As the test indicated a violation of the homoscedasticity assumption, heteroscedasticity-consistent standard errors (HC3) were used to ensure robust inference. While data were collected from 11 nursing facilities, we did not apply multilevel modeling. Following guidelines for small cluster designs (36), we treated observations as independent given the small cluster sizes (2–5 participants per facility), which limits reliable estimation of intracluster correlation coefficients and reduces the likelihood of substantial clustering effects. Analyses of the log data were conducted in R (37) using RStudio (38).

## Results

Eleven nursing homes, including 37 employees (31 females, 84%) and 137 residents (111 females, 81%), took part in this study.

### Nursing home residents

During the intervention period, six of the 137 residents passed away, and two moved out of their nursing home. The mean age of the residents was 85.0 years (SD = 7.6), and the mean body mass index (BMI) was 28.1 (SD = 5.8). Residents exhibited moderate cognitive impairment (mean MoCA score = 13.9 ± 6.6) and reduced functional mobility (mean TUG time = 27.6 ± 11.7 s), while digital competence was generally low (mean score 1.9 ± 1.1 on a 5-point Likert scale). Sample characteristics are presented in Table 1. Residents reported a mean continuance intention score of 3.9 (SD = 1.1). The perceived influence of the gamification feature on motivation to participate in the program was at 1.8 (SD = 1.2). Results on continuance intention and the motivational impact of the gamification feature are shown in Table 2.

**Table 2:** Results on continuance intention and motivational impact of gamification feature (nursing home residents).

Variable	*N*	Mean (SD)
Continuance intention	108	3.9 (1.1)
Influence of the gamification feature on motivation	81	1.8 (1.2)

**Table 1:** Subject characteristics (nursing home residents).

Variable	*N*	Mean (SD)
Age	137	85.0 (7.6)
Female, N (%)	137	111 (81.0)
BMI	137	28.1 (5.8)
Cognition (montreal cognitive assessment)	132	13.9 (6.6)
Digital competence score	134	1.9 (1.1)
Timed up and go test (time in sec)	95	27.6 (11.7)

### Nursing home employees

The employees involved in the BeSt Age program included 32 activity coordinators, four administrative personnel, and one occupational therapist. The mean age of nursing home employees was 51.7 (SD = 11.5) years. Twenty reported having a certification related to instructing physical activity, and mean years of experience delivering physical activity were 7.9 (SD = 7.2). Employees rated their digital competency as good (3.54; SD = 0.8). The characteristics of the participating employees are presented in Table 3.

**Table 3:** Subject characteristics (nursing home employees, *n* = 37).

Variable	Mean (SD)
Age	51.7 (11.5)
Female, *N* (%)	31 (83.8%)
Job	Activity coordinator (32), Administration (4), Occupational Therapist (1)
Experience in delivering PA (years)	7.9 (7.2)
Digital competence score	3.5 (0.8)
Certification to deliver PA	20

Usability was rated at 87.1 ± 15 out of 100 points, and mean user experience score was 1.94 ± 1.49. Perceived usefulness yielded a mean score of 3.53 (SD = 1.05). Continuance intention was rated at 4.03 (SD = 1.22). An overview of the acceptance-related outcomes is provided in Table 4.

**Table 4:** Acceptance-related outcomes (nursing home employees).

Variable	*N*	Mean (SD)
Usability	32	87.1 (15.0)
User experience	30	1.94 (1.49)
Perceived usefulness	34	3.53 (1.05)
Continuance intention	35	4.03 (1.22)

The regression model (Table 5) explained 37.7% of the variance in continuance intention F(2, 28) = 10.06, *p* < .001, *n* = 31 (adjusted R² = .377), indicating a large effect size (39). Both usability (*β* = .46, *p* = .047) and perceived usefulness (*β* = .39, *p* = .020) were significant predictors of continuance intention.

**Table 5:** Results of the multiple regression analysis (*n* = 31).

Predictor	B	SE B	*β*	t	p
(Constant)	−1.15	1.79	–	−.64	.526
Usability	.04	.02	.46	2.08	.047
Perceived Usefulness	.44	.18	.39	2.46	.020

R² = .403, Adjusted R² = .377, F(2, 28) = 10.06, *p* < .001. HC3 robust standard errors were used.

### Log data

Employees delivered 85.6% of the predetermined sessions. Residents attended 75.1% of the provided sessions (adjusted adherence) and 64.3% of the planned 24 sessions (unadjusted adherence). Employee adherence was highest during weeks 1–4 (90.8%), followed by weeks 5–8 (83.7%) and weeks 9–12 (82.6%). Resident adherence was 65.5% in weeks 1–4, 66.6% (SD = 33.8) in weeks 5–8, and 60.8% (SD = 42.7) in weeks 9–12. Adjusted resident adherence was 72.2%, 79.6%, and 73.7%, respectively. Adherence results are displayed in Table 6. Adherence rates were additionally aggregated at the nursing-home level to describe between-site variability (Table 7). Across the 11 participating facilities, employee adherence ranged from 35.4% to 100.0% (median: 86.6%), resident adherence from 22.3% to 96.5% (median: 63.8%), and adjusted resident adherence from 62.4% to 96.5% (median: 70.7%). Reasons for non-adherence of residents were physical complaints (44%), overlapping appointments (such as dentist appointments, 19%), motivational reasons (12%) and other reasons (such as unexpected visits from family, 25%).

**Table 6:** Adherence results.

Variable	*N*	Overall M (SD)	Interval 1, M (SD)	Interval 2, M (SD)	Interval 3, M (SD)
Adherence (employees, in %)	11	85.6 (24.6)	90.8 (26.7)	83.7 (21.7)	82.6 (34.6)
Adherence (residents, in %)	129	64.3 (32.8)	65.5 (32.1)	66.6 (33.8)	60.8 (42.7)
Adjusted adherence (residents, in %)	129	75.1 (30.2)	72.2 (31.3)	79.6 (32.7)	73.7 (40.1)

Employee adherence was calculated at the nursing-home level (*n* = 11 facilities) because employees shared one tablet within each facility. Resident adherence and adjusted resident adherence were calculated at the individual level (*n* = 129 residents). Intervals correspond to weeks 1–4, 5–8, and 9–12 of the intervention period.

**Table 7:** Variability in adherence between facilities.

Variable	Min	Median	Max
Adherence (employees)	35.4%	86.6%	100%
Adherence (residents)	22.3%	63.8%	96.5%
Adjusted adherence (residents)	62.4%	70.7%	96.5%

All values are aggregated at the nursing home level (*n* = 11 facilities). Employee adherence reflects the proportion of planned sessions delivered per facility. Resident adherence and adjusted resident adherence are based on residents’ attendance, aggregated per facility.

Log data analysis according to the FITT-framework revealed that employees accessed the app, on average, on 34.7 days (SD = 10.9) of the total 84-day intervention period (frequency). The mean number of recorded screen touches was 3,436 (SD = 1,307) per nursing home during the whole intervention period (intensity). The mean duration between participants' first and final login sessions was 85.1 days (SD = 5.4) (time). Analysis of feature utilization (type) revealed that the personalization feature emerged as the most frequently accessed component, with a mean of 325 touches (SD = 262). The gamification feature was the second most frequently used component with a mean of 66 touches (SD = 43), followed by the customization feature with a mean of 46 touches (SD = 74). The health literacy feature was the least used component, averaging 14 touches per participant (SD = 17). Facility-level ranges indicated considerable variability across nursing homes for all FITT dimensions (Table 8).

**Table 8:** Log data analysis according to the FITT-scheme.

Variable	M (SD)	Min/Max
Frequency (days)	34.7 (10.9)	22/56
Intensity (touches)	3,436 (1,307)	1,682/6,418
Time (days)	85.1 (5.4)	77/90
Type: Personalization (touches)	325 (262)	25/899
Type: Gamification (touches)	66 (43)	24/109
Type: Customization (touches)	46 (74)	0/198
Type: Health literacy (touches)	14 (17)	0/61

Data collected from 11 nursing homes, each equipped with one tablet. Min/Max, minimum and maximum values across nursing homes.

## Discussion

This study examined the user acceptance of the BeSt Age App, a fall prevention and physical activity promotion mobile application for nursing homes, and analyzed the factors influencing employees’ continuance intention. A comprehensive understanding of the app's acceptance was derived from both subjective evaluations and objective usage data. The findings indicate that the application achieved high levels of user acceptance among nursing home employees, with perceived usefulness and usability significantly predicting continuance intention. This aligns with the TAM (13–15) and the Post-Acceptance Model of Information Systems Continuance (16), which highlights the central role of these constructs in shaping expectation confirmation and retrospective evaluations of technology.

The high usability score and positive user experience ratings for the BeSt Age App support the suitability for use in time-constrained care environments. With a usability score of 87 points, the BeSt Age App exceeds both the average SUS score of 76.6 for digital health applications overall and the mean SUS score of 83.3 for physical activity apps specifically, as reported by Hyzy et al. (40) in their meta-analysis. Usability emerged as a slightly stronger predictor than perceived usefulness, which may reflect the particular importance of intuitive design when implementing technology in healthcare environments. This finding suggests that even highly useful applications may face adoption barriers if their usability is insufficient. To this end, Park et al. (41) highlight the ongoing need for educational initiatives among nursing home employees, aligning with the premise that enhanced training can improve perceived ease of use. By providing employees with relevant knowledge and skills, nursing homes can cultivate a more favorable attitude towards technology and its usefulness in quality patient care. Similarly, Chisholm et al. (42) discuss the importance of organizational resources and management practices in fostering a culture of technology adoption. Their results suggest that increased allocations for training and development can positively shift employee attitudes toward effectively using technology in caregiving contexts.

The positive continuance intention score supports the app's potential for sustainable implementation in nursing home environments. While the moderate perceived usefulness score suggests that employees recognized the app's value, there may be room for enhancement in demonstrating clearer usefulness to users. This finding is consistent with user acceptance research, where perceived usefulness often requires time to fully develop as users gain experience with new systems and observe tangible outcomes (43).

The study revealed good implementation fidelity, with employees delivering most of the planned sessions. The interval-based analysis indicated that employee adherence was slightly higher in the first four weeks and showed a modest decline thereafter, which is broadly consistent with systematic review evidence showing that engagement with and adherence to mHealth interventions tends to decrease over time (44). Residents attended three-quarters of the sessions provided, however, the unadjusted adherence of 64.3% to predetermined sessions highlights the challenges inherent in implementing structured physical activity programs in nursing home settings. Comparable adherence rates have been reported in both analog (45–47) and digital interventions (48), suggesting that participation barriers persist across delivery modes. Reported barriers such as physical complaints and scheduling conflicts are consistent with prior findings (45). As noted by Vseteckova et al. (49), tailored, individualized and supported physical activity, led by a knowledgeable and well-communicating therapist, increases participation in group exercise among nursing home residents.

Analysis of log data revealed moderate but consistent involvement, with employees accessing the app on approximately 41% of available days. This finding stands in contrast to those reported by Amagai et al. (50), who found that only 9% of mobile health app studies successfully maintained participant involvement throughout their interventions. However, it is important to note that the studies included in their review primarily included individuals using an app for personal health purposes rather than in a professional caregiving context. Beyond involvement, the substantial variance in usage metrics suggests heterogeneity in engagement patterns across the 11 participating nursing homes. Notably, the customization and health literacy features were not used at all in some facilities while being used extensively in others. This variation suggests that facility-level factors such as organizational culture, staffing, and digital readiness may influence which app features are adopted in practice. Across facilities, however, the personalization feature was used more frequently than health literacy content, indicating a stronger practical demand for individualized intervention support than for educational information. The limited engagement with health literacy features suggests that educational content may require alternative presentation formats or integration strategies to enhance utilization. The observed mean continuance intention score indicates that residents demonstrated a favorable disposition toward continued participation in the implemented program. This is consistent with research demonstrating that nursing home residents, including those with dementia, recognize the benefits of structured activities (51). Sondell et al. (52) reported high or very high motivation in 61.0% of attended exercise sessions, with in-session motivation exceeding pre-session motivation, underscoring the importance of targeted strategies to enhance pre-session motivation.

The limited impact of the gamification feature on resident motivation suggests that conventional gamification approaches may be less effective for older adults with cognitive impairment. Gamification strategies need careful adaptation for older adult populations, as older individuals tend to prefer games that resemble real-life experiences or traditional formats such as card and board games, which are familiar to this demographic (53). However, it is important to note that residents only had access to the gamification elements when nursing employees showed them the digital garden, which might have limited impact on resident motivation.

The strong contrast between employee and resident digital competency scores (3.5 vs. 1.9 respectively) highlights the persistent digital divide affecting older adults in institutional care. While smartphone and internet usage among older adults has been steadily rising over time (54), this trend may be less pronounced among nursing home residents who often have additional barriers related to cognitive and physical impairments. Nursing home employees, by contrast, display relatively high digital affinity, as also reported by Barisch-Fritz et al. (55). These findings emphasize the importance of employee-mediated technology interventions in nursing homes, where employees serve as crucial intermediaries between digital health tools and residents.

This study has several limitations that should be considered. The study's duration of approximately three months may not have been sufficient to observe long-term adoption patterns or the full development of perceived usefulness as employees gained more experience with the application. Additionally, the sample included nursing homes that volunteered to participate in this study, which may limit generalizability to settings with lower technology readiness. Residents with very severe cognitive, neurological, or motor impairments were excluded, as they were unable to safely participate in the exercise program; consequently, findings may not be generalizable to the most vulnerable nursing home residents. Furthermore, while continuance intention was analyzed within the framework of established acceptance models, such as the TAM and the Post-Acceptance Model of Information Systems Continuance, these models primarily capture individual-level perceptions and do not incorporate the complex interplay between users, technology, and organizational context. Contextual factors specific to nursing home environments, such as staffing levels and organizational culture, were not measured in this study, yet are likely to play an important role in technology adoption and usage patterns. Although usability and perceived usefulness were selected *a priori* as theoretically central predictors, additional factors such as satisfaction, social influence, perceived workload, and compatibility with existing care routines may also affect continuance intention but were not directly assessed in the present study. The resident sample was characterized by significant cognitive and motor impairments, which may have influenced both adherence and the effectiveness of certain app features. Reliance on staff-reported adherence of residents may also introduce bias. Finally, engagement was measured only through log data, thereby capturing the behavioral dimension of engagement, while cognitive and affective aspects were not assessed. Future research should investigate longer-term adoption patterns, incorporate measures of cognitive and affective engagement and investigate links between app usage, resident outcomes, and long-term continuance intention. The CeHRes Roadmap 2.0 (56) provides a useful framework for this by integrating contextual and organizational factors that influence implementation. To support long-term integration of such tools, institutions should invest in structured onboarding, ongoing technical support, and integrate app use into regular staff workflows. Additionally, exploring adaptive gamification approaches specifically designed for older adults with cognitive impairment may improve engagement and motivation (57). Research examining the relationship between app usage data and clinical outcomes would strengthen the evidence base for perceived usefulness and support broader implementation efforts.

## Conclusion

The BeSt Age App demonstrated good user acceptance among nursing home employees, with usability and perceived usefulness emerging as predictors of continuance intention. Although resident adherence was influenced by health-related limitations, implementation fidelity and adherence rates were promising. These results support the feasibility of employee-mediated mHealth interventions in nursing homes and highlight the importance of designing human-centered, adaptable digital tools tailored to the needs of both staff and residents. The study contributes valuable insights for the development and implementation of technology-supported fall prevention and physical activity programs in long-term care settings.

## Data Availability

The raw data supporting the conclusions of this article will be made available by the authors, without undue reservation.
